# Spinal cord herniation: management and outcome

**DOI:** 10.1007/s00701-012-1365-6

**Published:** 2012-05-16

**Authors:** Rob J. M. Groen, Maarten H. Coppes, Berrie Middel

**Affiliations:** Department of Neurosurgery, University Medical Center Groningen, University of Groningen, PO Box 30.001, 9700 RB Groningen, The Netherlands

Dear Editor,

We read with interest the recent publication by Prada et al. [5] about the management and outcome of a series of 12 patients that were operated on for symptomatic thoracic spinal cord herniation.

Anterior transdural spinal cord herniation (ATSCH) has been recognised as a rare, probably under-diagnosed, but treatable cause of (progressive) thoracic myelopathy. It is typically a diagnosis of the MRI era, which has resulted in an increase in the number of publications over the past decade, comprising a total of approximately 170 cases to date. Clinical experience with this entity is limited. As a result, treatment strategies of ATSCH are based on individual cases and on the small series reported in the literature.

The authors’ surgical remedy consists of a posterior approach (laminectomy) with reduction of the herniated portion of the thoracic cord, and filling of the dura defect with a muscular plug, which is covered with a patch, and either sutured or “glued”, or both (suture and glue).

Dr. Prada and co-authors stated that they “attempt to establish treatment guidelines according to data obtained through *an extensive review of the literature*”. Their manuscript was submitted in August 2011, and accepted 3 months later. It is surprising that a significant number of very relevant recent publications which appeared in renowned neurosurgical journals before that date (i.e. the papers by Hassler et al. [2], Groen et al. [1], Imagama et al. [3], Shin and Krishnaney [6] and Nakamura et al. [4]) have not been taken into consideration and/or have escaped the attention of the authors.

The present report by Prada et al. adds another 12 patients to the international series, and should serve the readership to define the preferred treatment when facing a patient with ATSCH in their own neurosurgical practice. However, while scrutinising the “Results” section, a number of inconsistencies appear, which definitely confuse the reader:

1. On page 724 the authors write that ”all patients except two had a combination of sensory, motor and sphincter dysfunction”, but from Table 1 it can be deduced that only four patients had sphincter dysfunction.
2. With respect to surgical outcome, the text and Table 1 likewise are conflicting: on page 726 it is stated that “worsening of preoperative symptoms occurred in two patients”, “one patient became plegic immediately after surgery…”, and “a mild worsening of motor and sensory functions occurred in two other patients…” [5]. Thus, from the text it appears that the early postoperative outcome was worse in five patients (42%), but nothing as such is reflected in Table 1.
3. Long-term outcome, as reported in Table 1, is reported unchanged in six cases (50%), while six patients improved (50%). It remains unclear into which category the patient that “dramatically worsened” … “requiring implantation of a Soletra spinal cord stimulator 16 months after surgery” (see page 726 [5]) was scored!
4. Also, it is confusing how “outcome” relates to “early outcome”. Is “outcome” relative to “early outcome” or are both compared with preoperative neurological status? Why do the authors report early outcome and (late?) outcome as separate items?
5. Postoperative imaging (MRI to confirm proper reduction of cord herniation, realignment of the spinal cord, and reappearance of a CSF signal around the spinal cord) is not adequately reported and documented.

In the “Discussion” section (page 728), the authors stated that “the enlargement of the dural defect should not be considered a treatment for SCH alone” [5]. However, no arguments are given why such a procedure should be regarded as insufficient. On the contrary, in the literature available, there is evidence that enlargement of the dura defect is very effective. Most of the Japanese neurosurgeons who reported on ATSCH adhere this technique. In an Individual Patient Data (IPD) Meta-analysis of 126 case reports, spinal cord release and subsequent widening of the dura defect (WDD) were associated with the highest prevalence of motor function improvement after operative treatment of ATSCH [1]. The same study revealed that spinal cord release (CR) in general (CR in combination with direct suture of the dura defect, application of a patch over the defect, or simple WDD) resulted in postoperative motor function improvement in 68% of cases analysed or in stabilisation of the neurological deficit in 19% [1]. With these data in mind, the operative results in the series reported by Prada et al. (50% unchanged, and 50% improved, disregarding the conflicting text and Table mentioned above) ask for an explanation. Unfortunately, a critical evaluation of their own material is missing in this paper.

The authors promote the use of a muscle plug and glue as an essential part of the closing procedure. However, based on the results reported, we are less optimistic about the benefit of muscle and glue. It is known from previous papers that such material might cause adhesion and tethering of the spinal cord in the postoperative stage. This can be detected on postoperative MRI. In our opinion, especially in cases that did not improve, MR confirmation of adequate reduction of cord herniation, of realignment of the thoracic spinal cord into the anatomical position, and verification of the re-establishment of the normal CSF signal around the spinal cord, is essential. The need to check on these items is illustrated in Fig. 3 (page 726): the spinal cord seems in close contact with the ventral dura mater, while the posterior dura is very close to the dorsal aspect of the spinal cord. It would be interesting to see a gadolinium-enhanced T1-weighted MRI of this area, because the images depicted suggest tethering of the spinal cord. Unfortunately, a number that would refer to the corresponding patient is missing. Otherwise the reader could have checked in the Table whether this case had improved or not.

In rare disorders like ATSCH, large prospective series from single departments or from multicentre studies are not available, and evidence-based operative strategies are lacking because the limited number of cases will never meet the level of sufficient statistical power. Standardisation of case reports, however, would allow for better comparison of individual cases, and enable IPD Meta-analysis, and improve the rationale for treatment. For this purpose, a consistent and sound report, with a clear presentation of patient characteristics and (post)operative data, are essential. Dr. Prada and co-authors are to be commended for their effort to report their series of ATSCH, but it is unfortunate that certain essential items were left out and others are confusing. Clinicians should be encouraged to report new cases and to describe the details that are discussed, in order to improve the knowledge and the rationale of treatment in this rare and intriguing disorder.
